# Machine learning-based modeling of malachite green adsorption on hydrochar derived from hydrothermal fulvification of wheat straw

**DOI:** 10.1016/j.heliyon.2023.e21258

**Published:** 2023-10-20

**Authors:** Shadi Kohzadi, Nader Marzban, Yahya Zandsalimi, Kazem Godini, Nader Amini, Shivaraju Harikaranahalli Puttaiah, Seung-Mok Lee, Shiva Zandi, Roya Ebrahimi, Afshin Maleki

**Affiliations:** a Environmental Health Research Center, Research Institute for Health Development, Kurdistan University of Medical Sciences, Sanandaj, Iran; b Student Research Committee, Kurdistan University of Medical Sciences, Sanandaj, Iran; c Leibniz Institute for Agricultural Engineering and Bioeconomy, Max-Eyth-Allee 100, 14469, Potsdam, Bornim, Germany; d Department of Water and Health, Faculty of Life Sciences, Jagadguru Sri Shivarathreeshwara University, Sri Shivarathreeshwara Nagara, Mysuru, 570015, Karnataka, India; e Department of Environmental Engineering, Catholic Kwandong University, Ganeung, 25601, South Korea

**Keywords:** Adsorption, Malachite green, Hydrochar, Hydrothermal fulvification, Machine learning

## Abstract

This study investigated the efficiency of hydrochar derived from hydrothermal fulvification of wheat straw in adsorbing malachite green (MG) dye. The characterizations of the hydrochar samples were determined using various analytical techniques like SEM, EDX, FTIR, X-ray spectroscopy, BET surface area analysis, ICP-OES for the determination of inorganic elements, elemental analysis through ultimate analysis, and HPLC for the content of sugars, organic acids, and aromatics. Adsorption experiments demonstrated that hydrochar exhibited superior removal efficiency compared to feedstock. The removal efficiency of 91 % was obtained when a hydrochar dosage of 2 g L^−1^ was used for 20 mg L^−1^ of dye concentration in a period of 90 min. The results showed that the study data followed the Freundlich isotherms as well as the pseudo-second order kinetic model. Moreover, the determined activation energy of 7.9 kJ mol^−1^ indicated that the MG adsorption was a physical and endothermic process that increased at elevated temperatures. The study also employed an artificial neural network (ANN), a machine learning approach that achieved remarkable R^2^ (0.98 and 0.99) for training and validation dataset, indicating high accuracy in simulating MG adsorption by hydrochar. The model's sensitivity analysis demonstrated that the adsorbent dosage exerted the most substantial influence on the adsorption process, with MG concentration, pH, and time following in decreasing order of impact.

## 1 Introduction

Water contamination stemming from natural and synthetic compounds is a big challenge across the globe because they are harmful to living organisms and can accumulate in the tissues of plants and animals [1,2]. Human survival is highly dependent upon maintaining healthy and safe water resources. Hence, it is of great importance to remove substances contaminating from them. Malachite green is a triphenylmethane and cationic dye; it is wildly applied in textile industry and in aquaculture activities as a fungicide, bactericide and pesticide. The high concentrations of this dye in aqueous environments can result in great adverse effects like carcinogenicity, mutagenicity, chromosomal fractures and teratogenic and respiratory toxicity on human [3,4]. It should be pointed out that there is an increasing worry about environmental and health problems; thus, new and beneficial processes are needed to well deal with these contaminants [5]. Different methods have been tested, but adsorption has been introduced as a fast and cost-effective process. Of course, adsorbent type is a key factor in the removal efficiency of the process [6,7]. Activated carbon, biochar, and hydrochar are carbon-based adsorbents, which benefit from advantages like high efficiency and cost efficiency. These materials can remove organic and inorganic compounds from water and wastewater [1]. In comparison with activated carbon and biochar, hydrochar need less energy and their production is easier [[8], [9], [10]].

Hydrochar is a carbonaceous material known for its high carbon content, which is produced through the hydrothermal carbonization (HTC) process of various biomass sources, including agricultural residues and animal wastes [11]. Hydrochar has advantages such as functional groups, porous structure, and high organic carbon, which make it a suitable substitution for a huge number of usages [11]. Furthermore, it has been documented that hydrochar is capable of removing several contaminants from aqueous solutions [12,13]. It is worth mentioning that the type of raw materials and the process conditions of HTC can influence the effectiveness of hydrochar as an adsorbent for pollutant removal [14,15]. The optimization of operating parameters, including temperature and reaction time, is of great importance in the HTC process, thereby enhancing the adsorption capacity of the resulting product [16]. Of course, to improve the removal efficiency of hydrochar for some pollutants, its modification during the process is essential [17]. Moreover, in order to modify hydrochar's surface, some chemicals and magnetic particles have been utilized [18]. It should be noted that porous structure and the surface functional groups can be changed by means of hydrochar surface modification. Consequently, the adsorption capacity of hydrochar enhances [19]. As such, hydrochar structural modification has become an important method for the development of its environmental applications [7]. Most of the methods used for this purpose are relatively complex and costly. Therefore, further research is needed to develop simple and cost-effective methods for hydrochar modification using modifiers [6], particularly in the case of anionic contaminants that do not practically tend to be adsorbed by these substances. It appears that modification through cheap alkalis, in addition to increasing the surface area and hydroxyl groups of hydrochar, is very cost-effective [7].

The HTC process leads to generating a liquid phase containing unreacted moieties such as aromatics, thereby decreasing the applicability of hydrochar for agricultural and environmental aims [20]. Recently, hydrothermal humification (HTH), and hydrothermal fulvification (HTF) have been applied to overcome this problem; the effluent of these processes can even be used for agricultural purposes. This processes leads to a liquid phase containing high amount of humic and fulvic acids. In comparison with the HTC process, the content of aromatics is reduced significantly in the liquid phase by using HTH, and HTF [21]. Tkachenko et al. identified the boundaries between the carbonization, humification, and fulvification processes. They reported that when the initial base amount ranges between 1 and 2 eq. of KOH to carbohydrate content, the process falls within HTH. However, with a further increase in the base content (more than 2 eq.), the process shifts towards HTF, leading to the breakdown of biomass into a more oxygenated homogeneous colloid. The hydrochar produced by HTH has been reported to act as a beneficial adsorbent [22].

Developing a cost-effective adsorbent involves predicting optimal operational conditions for efficient dye removal. Factors like concentration of dye, duration, pH level, the amount of adsorbent, temperature, and mixing velocity impact efficiency of adsorption and require prediction for effective adsorption [23]. While regression models for these operational parameters can be complex [24]. Recent research has turned to artificial intelligence, particularly artificial neural networks (ANNs) for prediction of adsorption efficiency. For example, a multilayer feed-forward neural network was used by Tanzifi et al. for modeling of methyl orange adsorption onto a polyaniline nano-adsorbent [25], and another study applied an ANNs to predict the removal of methylene blue on sodium alginate-kaolin beeds [23]. Both studies showed a high level of consistency between the results obtained through experimentation and those predicted. affirming the usefulness of ANN models for forecasting dye removal efficiency.

According to the above, in this study, a new type of hydrochar with suitable adsorption properties for adsorption malachite green was produced using HTF process. Hydrochar was produced using wheat residue because the amount of wheat production in Iran is about 13.5 million tons annually as announced by the Food and Agriculture Organization (2017). Therefore, a remarkable amount of wheat residue will be produced annually, and the feasibility of its use can be examined if this study is successful in the removal of pollutants [26]. The available literature lacks studies related to the adsorption of malachite green, a specific pollutant, using mentioned adsorbents. Therefore, the main objective of this study was to examine the adsorption process of MG from water utilizing hydrochar derived from the hydrothermal fulvification of wheat straw. Additionally, the study analyzed the impact of different adsorption factors such as pH, adsorbent dosage, and initial MG concentration. In addition to the evaluation of adsorption process using kinetic and isotherm models, the modeling of malachite green removal was performed using an artificial neural network (ANN) approach. Finally, the sensitivity analysis on process variables was conducted.

## 2 Materials and method

### 2.1 Chemicals

The chemicals employed in this study were of analytical grade and readily accessible in the market; KOH was purchased from Merck, MG dye (C_25_H_26_N_2_O_4_) was from Fluka. In all experiments, deionized water was used. In addition, the chemicals required for characterization of biomass and hydrochar were purchased from Carl Roth.

### 2.2 Preparation of hydrochar

The wheat straw was sourced from Sanandaj, Iran. Fiber fractions (NDF, ADF, ADL) were performed based on VDLUFA (2012; methods 6.5.1, 6.5.2 and 6.5.3 [27] using the FIBRETHERM FT12 Automated Fiber Analyzer (C. Gerhardt GmbH & Co. KG, Germany). Subsequently, the hemicellulose, cellulose, and lignin were determined by subtracting NDF from ADF, ADF from ADL (acid detergent lignin), and ADL itself, respectively. Here, NDF and ADF represent, respectively, neutral and acid detergent fiber. The biomass was soaked in deionized water for a duration of 24 h to eliminate the contaminants, followed by 24 h drying at 60 °C and milled to 0.5–2 mm particles. Hydrochar was produced by mixing 5 g of wheat straw with 100 mL of a 1 M KOH solution, and then transferring the mixture to a stainless-steel reactor. It was heated (200 °C) for 12 h, followed by filtration and subsequent drying (60 °C) for a duration of 24 h. The hydrochar yield of 43.5 % was achieved, and the average particle size, measured through particle size analysis, was found to be 18.5 μm. In accordance with the findings of Tkachenko et al., in 2023, when the amount of KOH exceeded 2 eq., the process was classified as hydrothermal fulvification [21]. Here, the quantity of KOH added to the wheat straw was determined to be 4.2 equivalents (eq.) of KOH per carbohydrate content, which is reported in the subsequent results and discussion section.

### 2.3 Characterization

A Nicolet iS5 ATR-FTIR spectrometer (Thermo Scientific Inc., Madison, WI, USA) was used to record the spectra of the hydrochar and biomass. The surface morphology and particle size were examined using a scanning electron microscopy (Phenom ProX Desktop, USA). In order to determine the hydrochar and biomass's content of ash (Ash%), the samples underwent a two-step method. First, a 24-h treatment (105 °C) was performed to determine the total solids (TS%) of the sample. Subsequently, they were exposed to a 5-h treatment at 550 °C. The organic total solid (oTS%) was calculated as the difference between TS% and Ash%. Elemental analysis (CHNS) was conducted using a Vario El elemental analyzer (Elementar Analysensysteme in Hanau, Germany). Also, oxygen content (O%) was calculated by means of subtracting the percentages of carbon (C%), hydrogen (H%), sulfur (S%), nitrogen (N%) as well as ash (Ash%) from 100. The higher heating value of the hydrochar was calculated using the correlation developed in Ref. [16] as:

1 HHV(MJ/kg)=0.3853C+44.98/O

where C and O are measured values of carbon and oxygen using the elemental analysis.

The presence of aromatic compounds: HMF, furfural, catechol, phenol, guaiacol and cresol in the hydrochar was analyzed. The test was conducted by using high-performance liquid chromatography (HPLC) with a Dionex ICS 3000 system (Thermo Fisher Scientific Inc., USA). The system utilized a Knauer Eurospher C18 column, which was kept at a constant temperature (30 °C). And the mobile phase consisted of a mixture of water and acetonitrile, and it was dynamically adjusted through a multistep gradient during the measurement process. An Ultimate 3000 system by DIONEX was used to measure the content of sugars and organic acids. The reason for the measurement of organic acids, sugars, and aromatic compounds and the procedure for preparation of sample for the analysis of these compounds can be found in Ref. [21]. The inorganic content was measured using ICAP6300 Duo ICP-OES system from Thermo Scientific with the autosampler (ASX-520, CETAC Technologies). A Brunauer–Emmett–Teller (BET) method (Micromeritics TriStar II, USA) was applied to measure the BET surface area of the samples via N2 at −196.15 °C.

### 2.4 pH_ZPC_

In order to determine pH_ZPC_, the process was initiated by preparing five separate beakers, each containing a 100 mL aliquot of a 0.01 M NaCl solution. Then the level of pH was adjusted in these beakers across a range from 3 to 11 using NaOH and HCl. Subsequently, 0.1 g of the hydrochar was introduced into each beaker, which was sealed with parafilm, and shaken for a duration of 24 h. Then, the solution's final pH was plotted versus initial pH to dermine the pH_ZPC_ [[28], [29], [30]].

### 2.5 Adsorption experiments

Malachite green (MG) adsorption was investigated by pH, Hydrochar dosage and MG concentration at fixed room temperature during 90 min. The batch experiments were conducted in 250-mL beakers, each of which contained 100 mL of MG solution. These beakers were placed on an orbital shaker rotating at 120 rpm. An UV–Vis spectrometer (DR 5000, Hack, Ca) was used for determination of MG concentration at 624 nm. For pH adjustment, NaOH and HCl were employed. At scheduled time intervals, samples were collected and then filtered through fiberglass. Prior to spectrometry analysis samples were centrifuged for 10 min. At the same experimental conditions, a MG sample without hydrochar also included.

A few isotherms: Langmuir, Freundlich, Dubinin Radushkevich and Temkin were employed and discussed in terms of their linearized equations to describe the equilibrium. Also, the kinetic of experiments were checked.

The dye removal efficiency was calculated using following equation:

2 $$Removal\left(\%\right)=\frac{C_{0}-C_{t}}{C_{0}}\times 100$$

### 2.6 Artificial neural networks

**Fig. 1:**
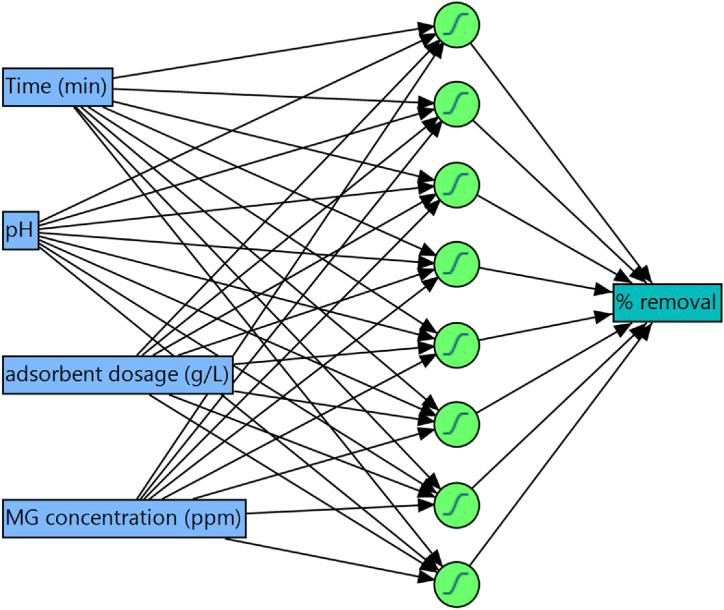
Neural network structure, including an input layer with 4 parameters, a hidden layer with 8 neurons, and output layer, which is the removal efficiency.

Artificial neural networks (ANNs) have demonstrated their effectiveness in discovering non-linear relationships between adsorption efficiency and the variables that govern the process [31]. ANNs are composed of several layers, including an input layer representing process variables (e.g., contact time, MG concentration, solution pH, and adsorbent dosage), hidden layers, and an output layer denoting adsorption efficiency (Fig. 1). Here, the JMP 17 pro software from SAS was used to develop a model for prediction of MG adsorption efficiency, following the procedure outlined in Ref. [23]. The Holdback method was used to randomly divide the data into training (49 samples, 80 %) and validation (12 samples, 20 %) subsets.

Here, we employed ANN modeling approach with TanH activation for hidden layers and including the transform covariates, as a fitting option to enhance adaptability. To mitigate overfitting concerns, the weight decay' (L2 regularization) was introduced as a penalty method and the model performance was monitored through a validation dataset. Incorporating these refinements, coupled with an adjusted number of hidden layers, demonstrate our approach to enhancing model performance, as evidenced by an R^2^ of 0.98 for the test set and 0.99 for the validation set (Table 1). Total number of 8 neurons were selected as the optimal number for the hidden layer. This choice was determined by the low RMSE (3.81) and the high R^2^ (0.98) achieved with the training dataset, which resulted in a similar model performance with the dataset used for validation (RMSE of 3.13 and an R^2^ of 0.99).

**Table 1:** RMSE and R^2^ for different umber of neurons in hidden layer.

Number of neurons	Stage	Sample	RMSE	R^2^
3	Training	49	4.40	0.98
	Validation	12	9.91	0.85
4	Training	49	8.10	0.92
	Validation	12	13.55	0.73
5	Training	49	5.12	0.97
	Validation	12	4.40	0.97
6	Training	49	3.36	0.99
	Validation	12	4.71	0.97
7	Training	49	4.24	0.98
	Validation	12	5.30	0.96
8	**Training**	**49**	**3.81**	**0.98**
	**Validation**	**12**	**3.13**	**0.99**
9	Training	49	5.86	0.95
	Validation	12	4.60	0.98
10	Training	49	5.12	0.97
	Validation	12	3.50	0.99

## 3 Results and discussion

### 3.1 Biomass and hydrochar properties

The fiber analysis indicated that the biomass had high cellulose and hemicellulose, but low lignin content (Table 2).

**Table 2:** Fiber analysis of raw wheat straw.

NDF%	ADF%	ADL%	Cellulose	Hemicellulose	Lignin
81.16	44.15	3.99	40.16	37.01	3.99

In the hydrothermal carbonization (HTC) process, high cellulose and hemicellulose content usually results in a final acidic process liquid due to the formation of organic acids, which reduces the pH and catalyzes the formation of furans from sugars. The furans then condense into micro/nano spheres on the surface of the non-dissolved solid in the HTC process. This, together with migration of inorganics from the solid into liquid phase, can result in an increase of the HTC hydrochar carbon content compared to its biomass. In contrast, here in hydrothermal fulvification, which was performed under high starting pH, the carbon content of hydrochar was decreased compared to its biomass (Table 3).

**Table 3:** Elemental analysis, HHV (MJ/kg), and BET surface area of the biomass and hydrochar.

	C%	H%	N%	S%	O%	Ash%	H/C	O/C	HHV (MJ/kg)	BET (m^2^g^−1^)
**Wheat straw**	44.9	6.89	0.16	0.11	40.86	7.08	1.84	0.68	18.4	6.51
**Hydrochar**	35.14	3.61	0.01	0.16	17.72	43.36	1.23	0.38	16.08	4.80

**Fig. 2:**
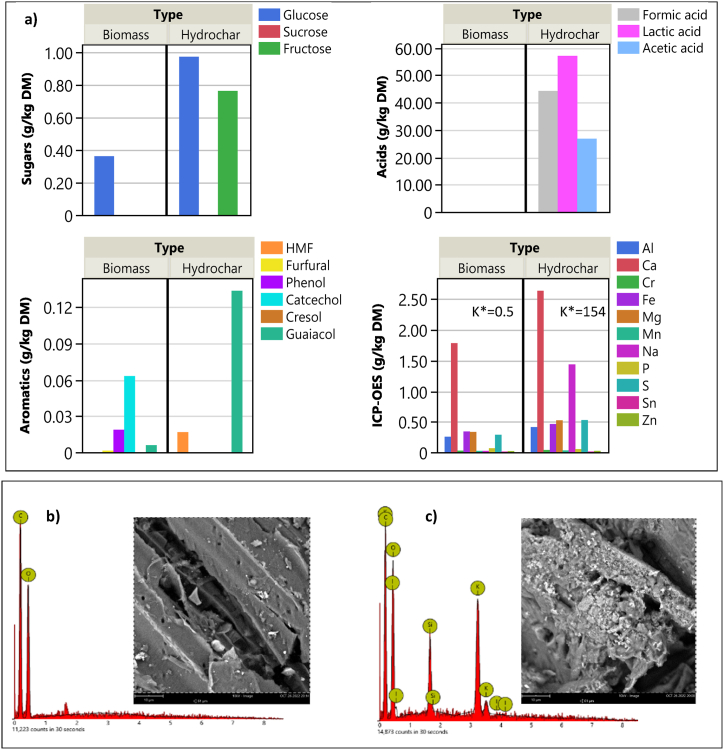
a) Concentration of sugars, organic acids, and aromatics and ICP-OES elements in biomass and hydrochar, b) SEM-EDAX analysis of biomass, and c) hydrochar. K* is potassium concentration in (g/kgDM) measured by ICP-OES method.

The fulvification process is evidenced by the conversion aromatics into humic and fulvic acids. Humification reactions has been observed in the liquid phase (with slightly high pH) after separation from the hydrochar and during storage [20]. In addition, a higher amount of lactic acid in the hydrochar compared to formic and acetic can prove the fulvification mood of the process, in which the hydrochar was produced (Fig. 2a). In comparison to previous studies on using HTC with neutral or acidic pHs [32,33], the solid product of the fulvification process has been found to have lower carbon content [21]. The lower HHV of hydrochar compared to the raw biomass is directly related to its lower carbon content [16], and a decline in the ratios of H/C and O/C indicates the removal of hydrogen and oxygen, making hydrochar more condensed. Furthermore, the high ash content of hydrochar resulted from the formation of an organic-inorganic complex structure through by using potassium hydroxide, as demonstrated by the ICP-OES analysis presented in Fig. 2. The hydrochar exhibits a significantly elevated potassium content (K = 154 g/kgDM) in contrast to the minimal potassium content found in raw wheat straw (K = 0.5 g/kgDM). The increase in ash content in hydrochar, derived from both the biomass and, particularly, the use of KOH during hydrothermal humification, contributes to a reduction in the BET surface area, leading to a less porous hydrochar. In future studies, investigating methods to improve the porosity and surface area of hydrochar, such as high-temperature pyrolysis, holds promise for further optimizing its properties and applications.

In Fig. 2(b and c), the SEM images and EDAX analysis indicate an absence of spherical solids. Instead, the surface of the hydrochar is predominantly covered with potassium.

**Fig. 3:**
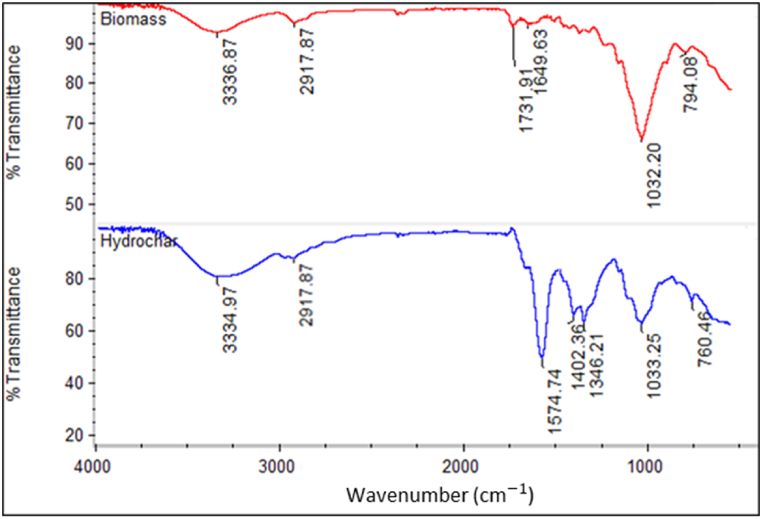
FTIR spectra of biomass and the hydrochar.

Fig. 3 displays the FTIR spectra of the biomass and hydrochar. The stretching vibrations of –OH groups cause a broad absorption band between 3330 and 3400 cm^−1^, which is partially derived from adsorbed water. The peaks at approximately 2917 cm^−1^ are because of the aliphatic stretching vibrations of C–H groups groups, which are more prominent in hydrochar. The hydrothermal process with a higher amount of base releases more aliphatic chains from the biomass structure. The peaks at 1574 cm^−1^, 1402 cm^−1^ in FTIR spectra of hydrochar are likely because of the presence of functional groups such as aromatic C=C stretching, C–H bending, respectively. The existence of these functional groups in hydrochar may have the potential to improve the adsorption of cationic dyes like MG by providing binding sites for interactions between the hydrochar and the dye molecules. Another peak of interest is at 1346 cm^−1^, which corresponds to hydroxyphenolic groups. These groups usually originate from hydrolyzed lignin surface compartments. At high levels of KOH, the reaction mixture avoids acidic conditions and these groups do not participate in acid-catalyzed condensation.

**Fig. 4:**
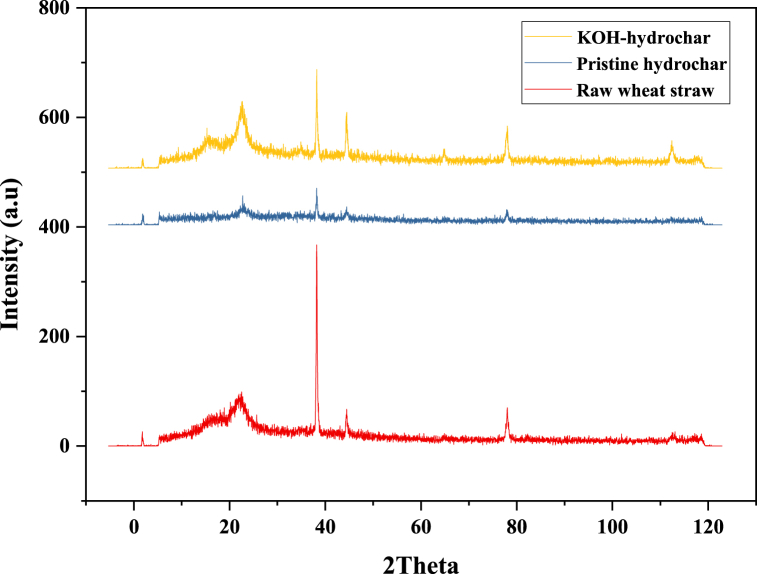
The XRD diffraction of biomass, hydrochar (no KOH) and hydrochar produced in hydrothermal fulvification process (KOH modified hydrochar).

To gain insights into the structural alterations occurring during the Hydrothermal Carbonization (HTC) process, X-ray diffraction (XRD) patterns of biomass, hydrochar (no KOH), and hydrochar from hydrothermal fulvification (here called KOH-modified) were analyzed (see Fig. 4). Broad peaks in the 2θ range of approximately 15–18 are characteristic of amorphous, non-crystalline constituents within biomass materials, including lignin and amorphous cellulose [34]. Additionally, the presence of peaks at 2θ 22 corresponds to the cellulose I crystal planes, specifically with Miller indices 110 and 002, signifying the presence of a crystalline cellulose structure [35].

Furthermore, the appearance of peaks at 2θ 38 and 45 may also be indicative of crystalline cellulose content. However, after subjecting the hydrochars to KOH modification, these cellulosic crystalline peaks exhibit reduced intensity, approaching a flatter profile and eventually disappearing. This pronounced decrease in crystallinity is particularly evident when comparing the KOH-modified hydrochar to the raw wheat straw, as evidenced by the broader and lower-intensity peaks observed in the former. Such observations strongly suggest a transformation of the material toward amorphous structures.

### 3.2 Adsorption of MG

#### 3.2.1 Impact of process parameters on adsorption

In this section, we present the influence of different parameters of adsorption process, such as pH, dosage, and MG concentration at different time intervals, on the adsorption of MG.

##### 3.2.1.1 Effect of pH and pHzpc

**Fig. 5:**
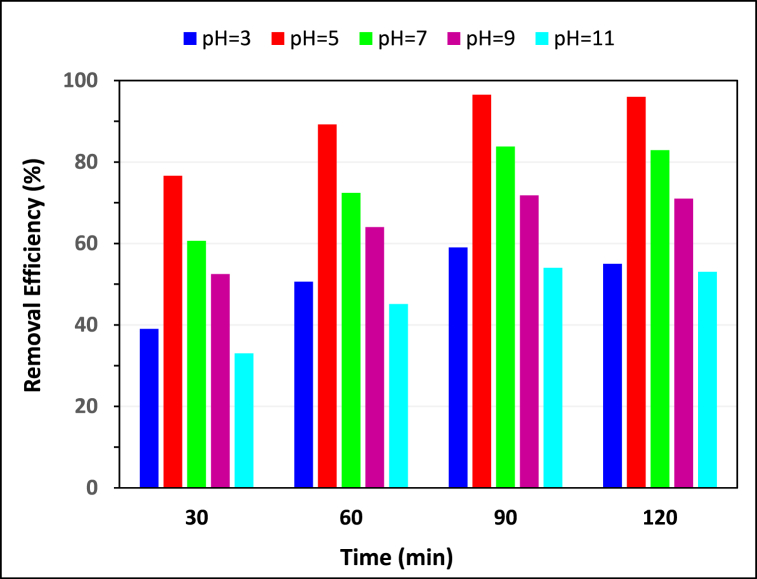
The effect of pH on the MG removal efficiency by hydrochar (MG = 20 mg L^−1^, KOH modified hydrochar = 2 g L^−1^).

Considering that pH has a direct impact on both the ionization of the dye and the surface charge of the adsorbent, it is consider as an effective factor that significantly impacts the optimization of the adsorption process [28]. The effect of pH on MG adsorption was explored within a range of 3–11; meanwhile, in all the experiments, the MG concentration and hydrochar dosage remained constant at 20 mg L^−1^ and 2 g L^−1^, respectively. As shown in Fig. 5, the removal efficiency of MG significantly rose from 55.22 to 96.62 % as the pH was raised from 3 to 5. But with increasing pH the removal efficiency decreased in a way that in the pH of 11 removal efficiency decreased to 53.11 %. Malachite green is a cationic dye and at low pH produced H^+^ will compete with the dye for occupancy of the surface active sites of the adsorbent. Based on the acid-base titration method, the pH_ZPC_ of the hydrochar was determined to be around eight, indicating a negatively charged surface at higher pH levels, which should enhance the removal efficiency of cationic substances like MG. However, this finding contradicts our experimental results. Decreasing adsorption in higher pH may attributed to producing soluble hydroxyl complexes [29].

##### 3.2.1.2 Effect of the hydrochar dosage

**Fig. 6:**
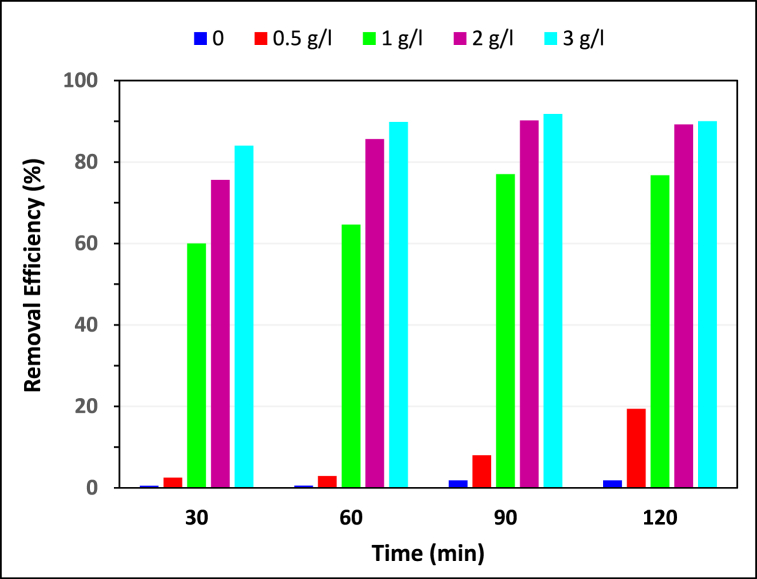
The effect of hydrochar dosage (g L^−1^) on the removal efficiency of MG (pH = 5.6 and MG = 20 mg L^−1^).

In order to optimize the adsorbent dosage for removal of MG, different hydrochar dosages ranging between 0.5 and 3 mg L^−1^ were tested at pH value of 5.6 and MG concentration of 20 mg L^−1^. Furthermore, to investigate the impact of time on MG concentration, a control sample was tested without the presence of an adsorbent. As shown in Fig. 6, the MG removal efficiency without the adsorbent over 120 min was only 1.8 %, illustrating minimal MG degradation over time. The removal efficiency has increased from 19 % to 89 % with increasing adsorbent dosage between 0.5 and 2 g L^−1^ because the adsorbent's superficial active sites has increased with increasing dosage [36] but remains constant (89 %) with increasing up to 3 g L^−1^. Hence, 2 g L^−1^ was selected as the optimum dosage. It should be noted that in the first 60 min of the experiment, most of the adsorption process has been done and with continuing up to 120 min the removal efficiency increased slightly.

##### 3.2.1.3 Impact of MG content

**Fig. 7:**
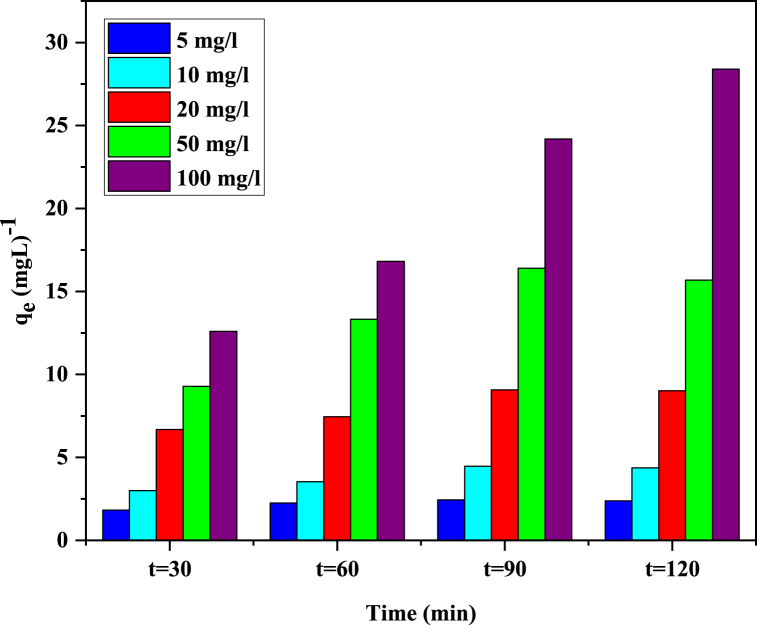
The effect of KOH modified hydrochar = 2 g L^−1^, pH = 5.6 on the removal of different MG concentrations.

For the purpose of determining the effects of MG content on the adsorptive removal capacity of hydrochar, experiments were designed at various MG concentrations (5–100 mg L^−1^). The removal efficiency with increasing dye concentration from 5 to 20 was slightly decreased from 96 to 90 % respectively however, removal efficiency shows a drastic diminish 61 and 54 % after escalating dye concentration to 50 and 100 mg L^−1^, respectively (Fig. 7). It owns to occupying available active sites on the surface of hydrochar with MG molecules.

Table 4 provides a comparison of the removal efficiency of MG and the maximum adsorption capacity of hydrochar produced via the HTF process with the findings of other adsorbent studies. Indeed, the hydrochar utilized in this study exhibits substantial adsorption capacity and exceptional removal efficiency, surpassing other investigated adsorbents.

**Table 4:** Comparison of the removal efficiency of the dye and the maximum adsorption capacity from aqueous solutions by hydrochar and different adsorbents.

Adsorbent type	Preparation conditions	Dye	Adsorbent dosage (g L^−1^)	Time (hr)	Dye concentration (mg L^−1^)	Removal efficiency (%)	Adsorption capacity (mg g^−1^)	pH	Ref.
Apple pomace	–	MG	0.5	24	50	100	–	7.2	[37]
Wheat Straw	–	MG	0.5	48	50	48	–	7.2	[37]
Hydrochar from algal bloom residues	10 h at 200 °C	MG^a^	4	24	50	92.1	89.05	–	[38]
Hydrochar from Fe_3_O_4_- orange peel	8 h at 200 °C	BB^b^	2.27	0.5	6.08	99	10.49	7	[39]
hydrochar from FeCl_3_ wheat straw	6 h at 200 °C	RhB c	1	1.5	2.5–25	91	80	6	[17]
Biochar from Rice – Husk KMnO_4_	4 h at 180 °C	MG	1	3	10	92.28	24.93	7	[40]
Biochar from rice Straw- KMnO	4 h at 180 °C	MG	1	3	10	86.86	23.37	7	[40]
Wheat straw hydrochar from HTH process	12 h at 200 °C	MG	2	1.5	20	91	13.28	5.6	This work

a Malachite green.

b Brilliant black.

c Rhodamine B.

##### 3.2.1.4 Experiments for desorption and reusability

The desorption experiments indicated that in the first and second step 19.3 and 5.5 % of the adsorbed dye were desorbed, respectively.

#### 3.2.2 Equilibrium isotherms

The adsorbent's adsorption capacity was determined below:

3 $$q_{e}=\frac{\left(C_{0}-C_{e}\right)\times V}{W}$$

where q_e_ represents the quantity of adsorbate adsorbed by the adsorbent (mg/g), V shows solution volume (L), C_0_ and C_e_ show, respectively, the initial and equilibrium concentration of the adsorbate (mg L^−1^), and W shows the mass of the adsorbent (g).

##### 3.2.2.1 Langmuir

The formula of Langmuir isotherm is stated below:

4 $$1/q_{e}=\frac{1}{K_{L}\;q_{\max}}\times \frac{1}{c_{e}}+\frac{1}{q_{\max}}$$

where C_e_ shows the equilibrium dye concentration in the solution (mg L^−1^), and q_e_ shows the equilibrium dye concentration on the sorbent (mg/g), and q_max_ and K_L_ show Langmuir constants representing the capacity of the adsorbent monolayer or Langmuir adsorption capacity (mg/g) and Langmuir adsorption constant (L/mg), respectively [30].

**Fig. 8:**
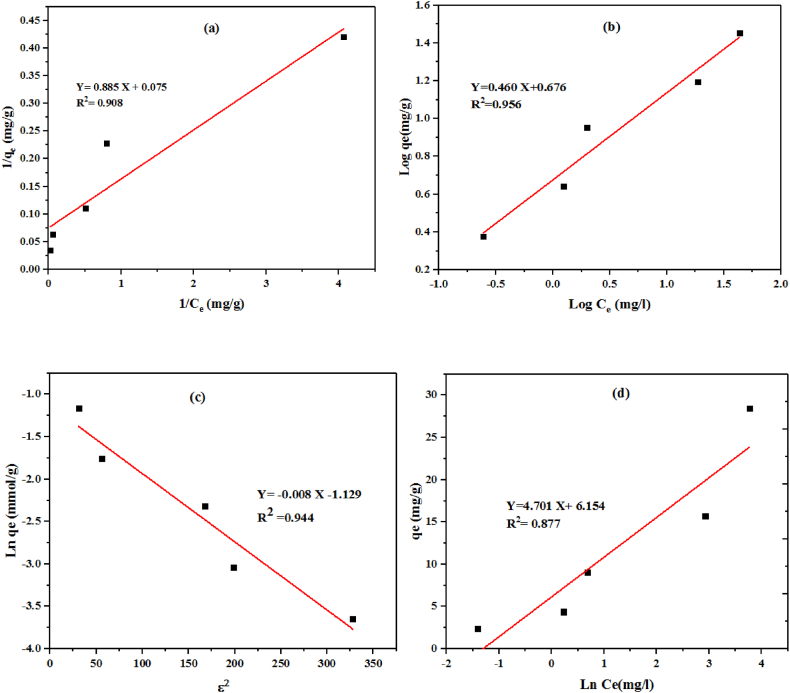
Linear form of a) Langmuir b) Freundlich model c) D-R and d) Temkin model model for MG adsorption.

The Langmuir isotherm model assumes that adsorption takes place as a monolayer on a uniform surface with identical adsorption sites. Each site has the capacity to hold the same number of adsorbed molecules, and there are no interactions between adjacent molecules. The k_L_ and q_max_ determined by plotting of 1/Ce versus 1/q_e_ as 0.85 L/mg and 13.28 mg/g, respectively (Table 5). Fig. 8 a shows that Langmuir equilibrium gives a good fit to the equilibrium adsorption data with R^2^ = 0.908. The adsorption capacity and R^2^ in this study are slightly higher compared to Chanzu et al. which was 1.484 mg/g and 0.704, respectively [41].

**Table 5:** The parameters of adsorption isotherm.

Models	Parameters	Value
Langmuir	q_max_ (mg g^−1^)	13.28
	K_L_ (L mg^−1^)	0.85
	R^2^	0.908
	R_L_	0.023–0.327
Freundlich model	K_F_	4.74
	n	2.16
	R^2^	0.956
D-R model	q_m_ (mg g^−1^)	117.99
	K_DR_	0.008
	R^2^	0.954
	E (kJ mol^−1^)	7.90
Temkin model	b_T_ (J mol^−1^)	4.70
	K_T_ (L g^−1^)	3.70
	B_T_	526.99
	R^2^	0.877

The dimensionless constant separation factor (RL) is a crucial parameter in the Langmuir isotherm that indicates the adsorption favorability. It is calculated below [42]:

5 $$R_{L}=\frac{1}{1+K_{L}C_{0}}$$

The R_L_ value is an indicator of the adsorption properties. Different ranges of R_L_ values correspond to different adsorption characteristics. An R_L_ value of 1 suggests a linear adsorption process following the Langmuir model. An R_L_ value between 0 and 1 indicates favorable and efficient adsorption. Conversely, R_L_ values exceeding 1 signify unfavorable adsorption, while an R_L_ value of 0 indicates irreversible adsorption with no expected desorption.

In the present study, for C_0_ between 5 and 100 mg L^−1^ the R_L_ ranged between 0.023 and 0.327. These ranges indicate using hydrochar for adsorption of MG can be considered favorable.

##### 3.2.2.2 Freundlich

In order to investigate the initial dye concentration data attained from equilibrium studies, the Freundlich equation was applied. The equation is conveyed as follows [43]:

6 $$\mathrm{Log}\;q_{e}=\mathrm{Log}\;K_{F}+\frac{1}{n}\mathrm{Log}\;C_{e}$$

Here, KF represents the Freundlich adsorption constant, and n represents the adsorption intensity. Their values can be obtained from the intercept and the slope of the graph when plotting Log qe against log Ce (Fig. 8b). In contrast with Langmuir model which describes adsorption on homogeneous surfaces, this model assumes a heterogeneous adsorption surface and active sites with divergent energy, besides it is not suitable at high adsorbate concentrations. Table 5 illustrates Freundlich values including adsorption capacity and intensity as 4.74 and 2.16. These results shows that Freundlich model has a lower adsorption capacity of MG onto hydrochar compared to Langmuir model.

##### 3.2.2.3 Dubinin Radushkevich (D–R)

The Dubinin–Radushkevich (D–R) model, which does not assume a homogeneous surface or a constant biosorption potential, is utilized to compute the mean free energy of adsorption per mole of the adsorbate (E, measured in kJ mol^−1^). This model also provides insights into determining the nature of adsorption, distinguishing between physisorption and chemisorption. The following equation represents the Dubinin and Radushkevich (D–R) equation [38]:

7 $$\mathrm{Ln}\;q_{e}=\mathrm{Ln}\;q_{m}-\left(\beta \varepsilon^{2}\right)$$

Also, β and *ε* are Dubinin Radushkevich (D–R) constants signifying coefficient related to the mean free energy of adsorption (mol^2^ J^−2^) and Polanyi potential (J mol^−1^), respectively.

As depicted in Fig. 8 c, the values of qm and β can be established by taking the intercept and slope, respectively, from the linearized isotherm plot, *ε* Vs Lnqe (measured in mmol/g). When the value of E falls within the range of 8–16 kJ mol^−1^, it suggests that the adsorption process is influenced by chemical ion-exchange. If the value of E is lower than 8 kJ mol^−1^, it indicates a physical adsorption process, while a value exceeding 16 kJ mol^−1^ signifies a chemisorption nature. In this case, the E value was determined to be 7.90 kJ mol^−1^, suggesting that the primary adsorption process for MG is physical in nature. The positive value of E indicates that the process is endothermic, meaning that higher solution temperatures would promote the sorption process.

##### 3.2.2.4 Temkin equilibrium

This model makes an assumption that the decrease in sorption heat is a linear process rather than logarithmic. The model additionally proposes that with an increase in adsorption layers and surface coverage, there is a linear decrease in the adsorption heat of molecules within those layers. This decrease is attributed to intermolecular interactions [44]. This model expressed as follows:

8 $$q_{e}=b_{T}\;\mathrm{Ln}\;K_{T}+\left(\frac{RT}{B_{T}}\right)\mathrm{Ln}\;C_{e}$$

where Ce shows the equilibrium concentration of the adsorbate, R stands for the gas constant (8.314 J mol^−1^K^−1^) and T shows temperature and held at 298 K. The parameters *b*_*T*_ and K_T_ show Temkin constants, where *b*_*T*_ represents the adsorption heat (J mol^−1^) and K_T_ shows the equilibrium binding constant (L/mg) [45]. The Temkin isotherm model was employed by plotting the q_e_ (mg/g) versus lnC_e_ (Fig. 8d). The value of *b*_*T*_ and K_T_ were calculated as 4.70 (J mol^−1^) and 3.70 (L/mg), respectively.

#### 3.2.3 Kinetic experiments

**Fig. 9:**
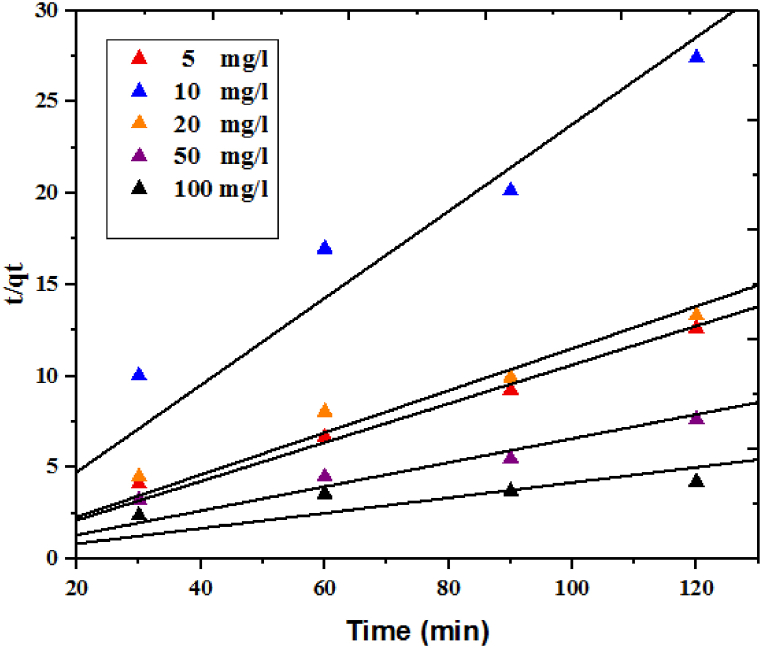
Pseudo-second-order kinetic models for the MG adsorption onto the hydrochar.

As indicated in Table 6 and Fig. 9, the R^2^ values of the pseudo-second-order model exceed those of the pseudo-first-order model for all dye concentrations. Besides, the theoretical q_e,cal_ calculated from the Pseudo second order kinetic is nearly equal to the experimental data (q_e,exp_). This observation validates the suitability of this model and the presence of chemisorption interactions in the adsorption process. It should be pointed out that the rapid phase observed in the initial 15 min may be attributed to physical adsorption or ion exchange occurring at the surface of the hydrochar. Therefore, it can be inferred that two distinct mechanisms were involved in the adsorption process (63).

**Table 6:** Kinetic fitting parameters for the MG adsorption by the hydrochar in different MG concentrations.

Dye concentration mg L^−1^	Pseudo second- order		
	R^2^	q_e_,_cal_ mg g^−1^	K_2_ mgg^1^min^−1^
5	**0.99**	**10.75**	**0.0077**
10	**0.97**	**5.42**	**0.0070**
20	**0.98**	**10.58**	**0.0048**
50	**0.97**	**21.09**	**0.0013**
100	**0.88**	**52.91**	**0.0001**

### 3.3 ANN modeling

**Fig. 10:**
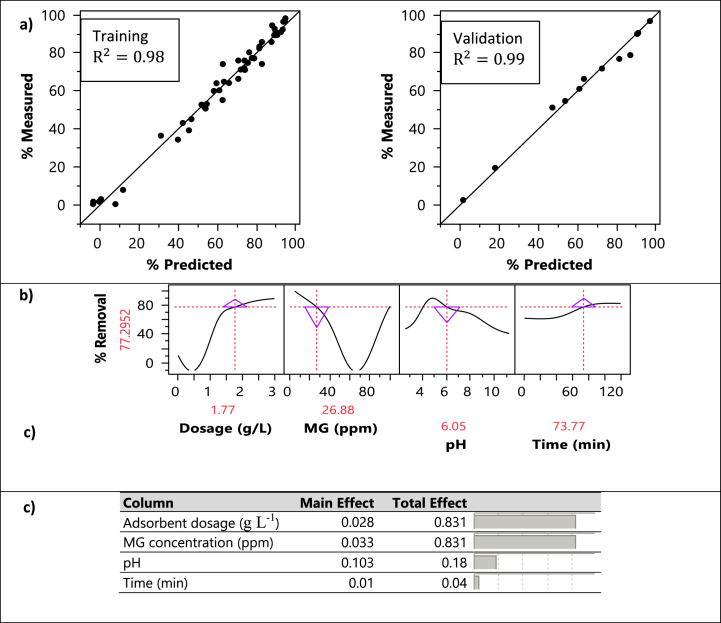
Results of ANN modeling as: a) actual by predicted removal plot, b) prediction profiler, c) variable importance.

In this section, an analysis and interpretation of the results obtained from the artificial neural network (ANN) model is presented. Fig. 10 shows key findings and insights derived from the model.

#### 3.3.1 Model performance evaluation

Fig. 10-a presents an evaluation of the ANN model's performance, developed in this study. Specifically, it displays a comparison between the actual experimental data and the model's predictions for both the training and validation datasets. As it was mentioned in material and methods section, an ANN model with 8 hidden layers was employed to develop a model for prediction of MG removal efficiency. The achieved high accuracy (R^2^ of 0.98 and 0.99 for the training and validation dataset), highlights the reliability of the developed model. This indeed shows a close agreement between the predicted and observed data, which confirms the model's suitability for MG adsorption prediction.

#### 3.3.2 Optimal adsorption conditions

The ANN model developed in this study, allowed us to identify the optimal conditions for MG adsorption on the adsorbent material. These conditions, depicted in Fig. 10-b, provide operational insights. The optimal adsorption parameters included hydrochar dosage: 1.77 g L^−1^, pH:6.05, initial MG concentration: 26.88 ppm, and contact time: 73.77 min.

#### 3.3.3 Sensitivity analysis

To enhance our understanding about the factors influencing MG removal, the concepts of main effect and total effect are shown in Fig. 10-c. The main effect analysis focusses the comparative contribution of each factor when studied separately. On the other hand, the total effect analysis reflects the effect of each factor on adsorption when considered alongside other factors. The results from this analysis reveal that the hydrochar dosage and MG concentration emerge as the factors with the most significant impact on predicted MG removal. This shows the importance of optimizing the quantity of adsorbent material, here hydrochar, and the initial dye concentration, to enhance adsorption efficiency. Additionally, the results show that the pH, and contact time are less effective compared to other factors on MG adsorption.

## 4 Conclusion

The experimental findings demonstrated that the production and alteration of the hydrochar and biochar derived from wheat straw result in an increased adsorption capacity. Additionally, HTH hydrochar, with its larger specific surface area, voids, and roughness, as well as smaller pore size, exhibited the highest adsorption capacity in comparison to other adsorbents. The adsorption process was impacted by the adsorbent dosage, initial MG concentration, pH and adsorption time and the adsorption capacity declined with raising dye concentration. The optimum removal efficiency of MG (91 %) was achieved at the concentration of 2 g L^−1^ hydrochar, MG concentration of 20 mg L^−1^ and pH of 5.6 in 90 min. The findings demonstrate that the reaction adheres to the pseudo-second-order kinetic model, and the data align well with the Freundlich isotherm model. This implies that under the applied experimental conditions, multilayer surface conditions may coexist. The successful application of the ANN with 8 neurons in its hidden layer (R^2^ = 0.98) was able to model the adsorption of MG by hydrochar. Upon performing sensitivity analysis on the model, it was found that the adsorbent dosage had the greatest impact on adsorption, followed by MG concentration, pH, and time in decreasing order of importance.

Additional research is advised to explore the production and modification of hydrochar derived from wheat straw as a potential adsorbent for pollutant removal.

## Ethical approval

“Not applicable”.

## Consent to participate

“Not applicable”.

## Consent to publish

“Not applicable”.

## Funding

“Kurdistan University of Medical Sciences, project no. IR.MUK.REC.1397/81.”

## Data availability statement

“Data included in article/supp. material/referenced in article”.

## CRediT authorship contribution statement

**Shadi Kohzadi:** Writing – review & editing, Writing – original draft, Investigation, Data curation. **Nader Marzban:** Writing – review & editing, Writing – original draft, Methodology, Conceptualization. **Yahya Zandsalimi:** Writing – review & editing, Investigation, Data curation. **Kazem Godini:** Writing – review & editing, Writing – original draft, Methodology, Investigation. **Nader Amini:** Writing – review & editing, Investigation. **Shivaraju Harikaranahalli Puttaiah:** Writing – review & editing, Conceptualization. **Seung-Mok Lee:** Writing – review & editing, Conceptualization. **Shiva Zandi:** Writing – review & editing, Investigation, Data curation. **Roya Ebrahimi:** Writing – review & editing, Investigation. **Afshin Maleki:** Writing – review & editing, Writing – original draft, Supervision, Methodology, Investigation, Conceptualization.

## Declaration of competing interest

The authors declare that they have no known competing financial interests or personal relationships that could have appeared to influence the work reported in this paper.
